# Professional values, ethical climate and job satisfaction of nurses and their selected sociodemographic and occupational characteristics

**DOI:** 10.3389/fpubh.2024.1501102

**Published:** 2024-11-05

**Authors:** Patrycja Ozdoba, Krzysztof Jurek, Beata Dobrowolska

**Affiliations:** ^1^Department of Holistic Care and Management in Nursing, Faculty of Health Sciences, Medical University of Lublin, Lublin, Poland; ^2^Institute of Sociology, Faculty of Social Sciences, John Paul II Catholic University of Lublin, Lublin, Poland

**Keywords:** sociodemographic, work environment, nurses, professional values, satisfaction, ethical climate

## Abstract

**Aim:**

To explore the relationship of selected socio-demographic and occupational characteristics of nurses and their level of professional values, hospital ethical climate and job satisfaction.

**Methods:**

Cross-sectional study was conducted among 388 Polish nurses from the spring of 2021 to winter of 2023, in the eastern part of Poland; and followed by Strengthening the Reporting of Observational Studies in Epidemiology (STROBE) guidelines. Four research tools were used to collect data together with questionnaire for socio-demographic and occupational characteristics.

**Results:**

Professional values such as activism correlate negatively with religious beliefs (*Z* = −1.789; *p* = 0.044), this means that nurses who are more involved in professional change activities are likely to be less associated with religious beliefs. A positive correlation was observed between the Ethical Hospital Climate Survey subscale—peer relations and nurses’ education level (*H* = 5.638; *p* = 0.048), indicating that a higher education level was associated with better relationships with colleagues at work. A negative relationship was identified between nurses’ external job satisfaction and their marital status (*Z* = −1.958; *p* = 0.040), that is, married nurses feel less satisfaction with the external aspects of their jobs than their single colleagues.

**Discussion:**

These findings underscore that medical staff management should take into account both sociodemographic factors [e.g., age, education, place of residence, marital status, religious beliefs, as well as professional factors (working hours, qualification course, etc.)] that affect nurses’ professional values, job satisfaction and the ethical climate of the hospital. These underscore the need to adapt management strategies to the individual needs of employees, which can contribute to improving working conditions in healthcare facilities.

**Data collection tool:**

The data collection tool consists of four sections.

**Demographics:**

Collected background and demographic information.

**Nurses’ professional values scale (NPVS-3):**

Assessed professional values among nurses.

**Hospital ethical climate survey (HECS):**

Assessed hospital ethical climate among nurses.

**Minnesota satisfaction questionnaire-short form (MSQ-SF):**

Assessed job satisfaction levels among nurses.

## 1 Introduction

Nurses’ professional values play a key role in shaping their attitudes, attitudes toward their work and the quality of the health care they provide (1). The professional values of nurses are the foundation of their daily work and include such aspects as empathy, responsibility, respect for patient autonomy, respect for patient dignity and the provision of professional care. It is also important to respect the principles of justice and to ensure the privacy and confidentiality of medical information. Nurses act with a high sense of responsibility and competence, constantly improving their skills, and collaborate with other members of the medical team to provide comprehensive and ethical care to patients, based on respect and understanding of their needs (2). Research is increasingly focused on identifying factors that may shape these values, taking into account both sociodemographic and work-related variables (3). Individual life experiences and long-term work in an occupation can influence occupational priorities and attitudes toward work. On the other hand, variables related to working conditions, such as workload, working hours and the type of duties performed, can also have a significant impact on the formation of nursing professional values (4). The work environment in which a nurses work can affect their sense of professional fulfillment and their willingness to pursue certain professional values (5). Understanding this relationship is important for both clinical practice and human resource management in the healthcare sector (6). In nursing practice, these values are reflected in daily decisions and actions, building an ethical climate that fosters responsible and empathetic care (6).

The ethical climate of a hospital directly affects the working environment of nurses, so ethical standards in the workplace are a foundation that affects the well-being and efficiency of nurses (7). Ethical climate, defined as a set of norms, values and practices for ethical behavior in the workplace, has a key impact on nurses’ behavior and decisions (8, 9). A positive ethical climate promotes decisions that are consistent with professional ethics, builds trust within the team, and increases job satisfaction (10). A negative ethical climate can lead to conflict, stress and professional burnout, which in turn reduces the quality of care provided (11).

Nurses’ job satisfaction is an important component of their professional well-being and has a direct impact on the quality of health care provided (12). In the face of increasing challenges in the healthcare sector, understanding the factors that influence job satisfaction becomes crucial (13). Research indicates that nurses’ job satisfaction can be analyzed in the context of a variety of variables, such as sociodemographic variables (e.g., age, gender, education) and variables related to working conditions (workload, quality of interpersonal relationships in the workplace, and opportunities for career advancement) (14, 15).

Consequently, understanding the relationship between nurses’ professional values, ethical hospital climate, job satisfaction, and sociodemographic and work environment variables is crucial to effectively managing nursing staff and creating a healthy, ethical work environment in healthcare facilities. A comprehensive approach to these relationships can help to develop strategies improving the well-being of staff and in a long-term the quality of care provided to patients.

## 2 Aim

To examine the relationship of selected socio-demographic and occupational characteristics of nurses and their level of professional values, hospital ethical climate and job satisfaction.

## 3 Materials and methods

### 3.1 Study design

In the period spanning from March 2021 to February 2023 within the Polish context, a descriptive, correlational, cross-sectional study was conducted. The research followed the Strengthening the Reporting of Observational Studies in Epidemiology (STROBE) guidelines, serving as a standardized framework for reporting observational studies (16).

### 3.2 Study participants and settings

The study involved 388 nurses who represented different levels of education: medical secondary school, BSN, and MSN. The study group was selected using a convenience sampling method. Inclusion criteria comprised: [1] voluntary participation consent, [2] possession of a valid nursing license, [3] current employment at a hospital in eastern part of Poland, [4] a minimum of 2 years of professional experience, and [5] availability of Internet access.

### 3.3 Research instruments

This study utilized four research instruments for data collection.

The tool assessing nurses’ professional values, known as **Nurses’ Professional Values Scale Three** (NPVS-3), was designed by Weis and Schank (17) based on research on the earlier NPVS and NPVS-R scales. It consists of 28 questions, divided into three subscales: caring, activism and professionalism. These questions assess nurses’ commitment to patient care, their initiatives in improving health care conditions, and their adherence to high ethical and professional standards. Respondents rate their answers on a 5-point Likert scale, and scores can range from 28 to 140 points, where higher scores indicate stronger professional values. In the original version of the NPVS-3, the Cronbach’s alpha coefficient was 0.94 (17), and in the Polish adaptation the value reached 0.95 (18).

The tool assessing the perceptions of nursing personnel regarding various facets of the ethical climate within their workplace, known as **Hospital Ethical Climate Survey (HECS)**. This survey tool, consisting of 26 questions rated by respondents on a 5-point Likert scale, where 1 signifies “almost never true” and 5 indicates “almost always true” (8, 9). The Polish adaptation comprises 21 items, with five items (Q1, Q2, Q4, Q8, Q9) removed due to not meeting psychometric criteria (19). HECS encompasses five subscales examining respondents’ interactions with peers, patients, managers, the hospital, and physicians. A higher total score reflects a more positive ethical climate within the surveyed organization. The original HECS version demonstrated a robust Cronbach’s alpha coefficient of 0.91 (7, 8), and for the Polish version, the coefficient stands at 0.93 (19).

The tool assessing the perceptions both intrinsic and extrinsic job satisfaction levels, known as **Minnesota Satisfaction Questionnaire-Short Form** (MSQ-SF). Intrinsic job satisfaction pertains to individuals’ perceptions of the inherent nature of their job duties, while extrinsic job satisfaction involves the evaluation of elements in the work situation beyond the specifics of job tasks (20). The MSQ-SF evaluates 20 job characteristics, utilizing a Likert scale ranging from 1 (I am not satisfied) to 5 (I am extremely satisfied). These characteristics encompass aspects such as achievement, independence, co-workers, recognition, and working conditions, as outlined by Weis in 1977. It’s important to note that the Cronbach’s *α* coefficient for the variables included in the Polish version of MSQ was reported as 0.86 (21).

The **Authors Original Questionnaire (AOQ)**, developed specifically for this study, included questions on the sociodemographic details of the participants (sex, age, marital status, place of residence, level of education, socioeconomic conditions etc.) and questions related to their work environment, including such elements as post-graduate education, form of employment, job position, work experience, working hours, age range of patients under care, additional workplace, etc.

### 3.4 Data collection process

The data collection spanned from March 2021 to February 2023 in Poland. In response to the limitations posed by face-to-face interactions during the COVID-19 pandemic, the decision was made to conduct the survey remotely, utilizing an online questionnaire created through the “Google Forms” platform. In our study, we focused on nurses from eastern Poland, using several methods to ensure that the participants were exclusively from that area. Recruitment involved sending email invitations to hospitals in the eastern Poland provinces, alongside disseminating information about the study on health science and nursing blogs, discussion platforms, and social media networks associated with nurses. Prospective participants received invitations with a link to the survey questionnaire and research instruments. In the questionnaire, we included questions regarding the profession and place of work to ensure that the data was collected solely from nurses in the designated area.

A total of 586 individuals initiated the questionnaire, with 388 (66%) successfully completing and returning it. The survey invitation communicated the purpose and step-by-step process of the study, emphasized the need for informed and voluntary consent, and provided contact details for the researcher to address any queries related to the study.

### 3.5 Statistical analysis

Statistical analysis was performed using IBM SPSS Statistics software, version 29. A descriptive approach was used to conduct statistical tests, which included estimating the frequency of distribution of variables, taking into account the standard deviation and mean percentage. As part of the evaluation, correlations between the NPVS-3, HECS and MSQ-SF scales, as well as sociodemographic and occupational variables, were tested using Pearson correlation, Mann–Whitney U test and Kruskal–Wallis test.

### 3.6 Ethical considerations

Participation in the study was entirely voluntary and anonymous. Before joining, participants were provided with detailed information about the study’s goals, processes, and characteristics, along with the freedom to withdraw from the study at any stage. It is worth noting that the Google Forms platform used to conduct the survey does not record the IP addresses of respondents, which further guarantees the anonymity of participants. All collected data is stored in an Excel file, secured with encryption, and each respondent is given a unique identification number, which ensures the confidentiality of the data. The researcher’s computer, on which this information is stored, is password protected, which is another safeguard against unauthorized access. The research commenced after obtaining approval from the Bioethics Committee at the Medical University of Lublin (reference number: KE-0254/17/2021). These measures were designed to guarantee participants full autonomy and safety in the research process.

## 4 Results

### 4.1 Study participants

A total of 388 nurses participated in the study, with an average age of about 41.60 years (SD = 11.52). The study showed that the respondents were predominantly women, accounting for as much as (*n* = 370, 95.4%) of the surveyed population, with a master’s degree in nursing (*n* = 207, 53.4%). Moreover, the majority of respondents rated their socioeconomic conditions as excellent (*n* = 192, 49.5%), while only (*n* = 16, 4.1%) considered them moderate. Detailed characteristics of the respondents are presented in Table 1.

**Table 1:** Sociodemographic characteristics of study participants.

Socio-demographic factors		*M*	SD
Years		41.60	11.52
		*n*	%
Sex	Female	370	95.4
	Male	18	4.6
Location of residence	Countryside	185	47.7
	Metropolitan area	203	52.3
Marital status	Living alone	97	25.0
	In a relationship (informal or formal)	291	75.0
Level of education	Medical secondary school /Medical vocational college	63	16.2
	BSN (Bachelor of Science in Nursing)	118	30.4
	MSN (Master of Science in Nursing)	207	53.4
Religious beliefs	Yes No		
Socioeconomic conditions	Excellent	192	49.5
	Fine	180	46.4
	Medium	16	4.1

The most popular place for nurses to work was in Anesthesiology and Intensive Care units, accounting for (*n* = 126, 32.5%) of the study group, while units such as Cardiology and Dermatology were represented to a lesser extent, (*n* = 3, 0.8%) and (*n* = 6, 1.5%), respectively. The majority of nurses (67.5%) did not have a specialty or were in the process of acquiring one. Working on a 12-h system was the most common, involving (*n* = 273, 70.4%) of nurses, in contrast to the 8-h system, which was practiced by (*n* = 68, 17.5%) of respondents. Employment based on an employment contract prevailed (*n* = 379, 97.4%), while a small number worked on a contract of mandate (*n* = 7, 1.8%) or as self-employed (*n* = 3, 0.8%). Detailed characteristics of the respondents are presented in Table 2.

**Table 2:** Characteristics of the work environment.

Work environment variables		*M*	SD
Job experience		20.60	11.52
Commuting time (min)		25.32	14.42
		*n*	%
Place of work (unit)	Anesthesiology and Intensive Care	126	32.5
	Cardiology	3	0.8
	Dermatology	6	1.5
	Geriatrics	14	3.6
	General Surgery	40	10.3
	Gynecology and Obstetrics	52	13.4
	Neurology	60	15.5
	Pediatrics	47	12.1
	Psychiatry	40	10.3
Life stage of patients under care	Neonatal/ infant	26	6.7
	After infancy/pre-school	21	5.4
	School age	19	4.9
	Adult age	308	79.4
	Old age	14	3.6
Nursing specialization	None or in progress specialization	262	67.5
	Post specialization	126	32.5
Taking a qualification course	Yes	196	50.5
	No	192	49.5
Taking a retraining course	Yes	198	51.0
	No	190	49.0
Working hours	8 h	68	17.5
	12 h	273	70.4
	Mixed	47	12.1
Job position	Nurse	55	14.2
	Senior nurse	311	80.2
	Coordinating nurse	8	2.1
	Deputy ward nurse	11	2.8
	Ward nurse	3	0.8
Form of employment	Employment contract	379	97.4
	Contract of mandate	7	1.8
	Self-employment	3	0.8

### 4.2 Nurses’ professional values and sociodemographic variables and work environment

The mean scores of the NPVS-3 subscales for care, activism and professionalism are, respectively: 24.23 (SD = 4.07), 39.17 (SD = 5.14) and 21.07 (SD = 3.68). The overall score of this scale was 84.47 (SD = 7.46). A negative correlation was observed between the dimension of care and two variables such as age (*r* = −0.178; *p* = 0.024) and participation in a retraining course (*Z* = −1.921; *p* = 0.045). The activism dimension correlates negatively with religious beliefs (*Z* = −1.789; *p* = 0.044), while the overall NPVS-3 scale score shows a positive relationship with nurses’ commuting time (*r* = 0.101; *p* = 0.047) (Table 3).

**Table 3:** Correlations between nurses’ professional values and selected sociodemographic and work environment variables.

Descriptive statistics			Variables							
Subscales	*M*	SD	Age		Religious beliefs		Retraining course		Commute time to work	
			*r*	*p*	*Z*	*p*	*Z*	*p*	*r*	*p*
Dimension of caring	24.23	4.07	**−0.178**	**0.024**	−1.269	0.204	**−1.921**	**0.045**	0.013	0.795
Dimension of activism	39.17	5.14	0.042	0.408	**−1.789**	**0.044**	−1.654	0.098	0.089	0.081
Dimension of professionalism	21.07	3.68	0.062	0.223	−0.222	0.824	−1.191	0.234	0.066	0.195
NPVS-3	84.47	7.46	0.017	0.739	−0.379	0.704	−1.474	0.141	**0.101**	**0.047**

*M, mean; SD, standard deviation; r, Pearson correlation coefficient; Z, Mann–Whitney U test. Bold - statistically significant correlation.*

### 4.3 Nurses’ job satisfaction and sociodemographic variables and work environment

The average scores for intrinsic satisfaction, extrinsic satisfaction and overall satisfaction (MSQ-SF) are: 24.42 (SD = 3.78), 35.95 (SD = 5.05) and 60.37 (SD = 6.29) respectively. A positive relationship was observed between the dimension of extrinsic satisfaction and age (*r* = 0.114; *p* = 0.025), job experience (*r* = 0.114; *p* = 0.025), education (*H* = 6.645; *p* = 0.036) and working hours (*H* = 5.993; *p* = 0.032). A negative relationship was additionally found between external job satisfaction among nurses and their marital status (*Z* = −1.958; 0.040) (Table 4).

**Table 4:** Relationships between nurses’ job satisfaction and selected sociodemographic and work environment variables.

Descriptive statistics			Variables									
Subscales	*M*	SD	Age		Job experience		Marital status		Education		Working hours	
			*r*	*p*	*r*	*p*	*Z*	*p*	*H*	*p*	*H*	*p*
Intrinsic Satisfaction	24.42	3.78	−0.066	0.193	−0.066	0.193	−0.813	0.416	3.948	0.139	0.666	0.717
Extrinsic Satisfaction	35.95	5.05	**0.114**	**0.025**	**0.114**	**0.025**	**−1.958**	**0.040**	**6.645**	**0.036**	**5.993**	**0.032**
MSQ-SF	60.37	6.29	0.052	0.308	0.052	0.308	−1.134	0.257	1.684	0.431	1.445	0.486

*M, mean; SD, standard deviation; r, Pearson correlation coefficient; Z, Mann–Whitney U test; H, Kruskal–Wallis test. Bold - statistically significant correlation.*

### 4.4 Ethical hospital climate among nurses and sociodemographic variables and work environment

The mean scores for the subscales on ethical hospital climate among nurses oscillate between 11.82 (SD = 2.74)—relations with peers and 17.93 (SD = 3.69)—relations with managers. A positive correlation was found between the subscale of peer relationships and nurses’ education (*H* = 5.638; *p* = 0.048). On the other hand, the relationship with physicians subscale correlates negatively with participation in the qualification course by nurses (*Z* = −2.706; *p* = 0.048) (Table 5).

**Table 5:** Correlations between ethical hospital climate and selected sociodemographic and work environment variables of nurses.

Descriptive statistics			Variables			
Subscales	*M*	SD	Education		Qualification course	
			*H*	*p*	*Z*	*p*
Relations with peers	11.82	2.74	**5.638**	**0.048**	−0.676	0.499
Relations with patients	12.08	2.77	1.437	0.488	−1.144	0.253
Relations with managers	17.93	3.69	0.405	0.817	−1.057	0.291
Relations with the hospital	17.87	3.46	2.261	0.323	−0.274	0.784
Relations with physicians	17.97	3.53	0.785	0.675	**−2.706**	**0.048**
HECS	77.66	7.27	0.201	0.904	−0.218	0.827

*M, mean; SD, standard deviation; Z, Mann–Whitney U test; H, Kruskal–Wallis test. Bold - statistically significant correlation.*

## 5 Discussion

Professional values, the ethical climate of the hospital as well as job satisfaction are key aspects of nurses’ experience of working that influence their commitment, motivation and the quality of their work. In this study, we analyzed selected socio-demographic and work factors in terms of their relationship with nurses’ professional values, ethical hospital climate and job satisfaction.

In our study, there was a negative correlation between the dimension of care—the professional value of nurses and age (*r* = −0.178; *p* = 0.024), where the same relationship was obtained among Polish nursing students (22). The negative correlation between age and dimensions of care suggests that younger nurses may place more importance on aspects of care in their work compared to older colleagues. This may be due to generational differences, where younger generations of nurses may be more motivated or more committed to providing care to patients (23). On the other hand, older nurses may have more professional experience, which may influence their broader view of their work, which includes other aspects beyond direct care (such as management, education of new personnel, and career development), contradicting the results obtained by researchers in Egypt (24). Professional burnout, which is a common problem among nurses with longer tenure, can lead to less commitment to patient care. Physical and emotional fatigue from years of working under stressful conditions can make older nurses less likely to engage in intensive patient care (25).

The negative correlation between participation in a retraining course and dimension of care (*Z* = −1.921; *p* = 0.045) may indicate that nurses attending courses must simultaneously fulfill their normal job duties, they may feel overworked, resulting in a decrease in the quality and level of commitment to care (26). Attending refresher courses can result in additional workload, which can limit the time and resources available to devote to patient care. As a result, they may assign less importance to aspects of care in their work (27).

The professional value of activism in nursing refers to the level of nurses’ involvement in initiatives to improve working conditions, raise the quality of health care, protect patients’ rights, and bring about systemic change at various levels of health care (28). Activism can include participation in professional organizations, engaging in community activities, promoting social justice in health care, and fighting for better working conditions for nurses (17).

The negative correlation between the level of professional activism and nurses’ religious beliefs (*Z* = −1.789; *p* = 0.044) means that there is an inverse relationship between the two variables. In other words, the more involved nurses are in professional activities, the weaker their religious beliefs may be, and vice versa. The relationship between religiosity and activism among nurses may seem surprising, since intuitively it might seem that religiosity affects the care dimension more than activism. There was a higher level of activism among religious respondents than among non-believers. A likely reason for the higher level of activism among religious respondents compared to non-believers may be that many religions place a strong emphasis on action for others and community involvement. Religious nurses may be more motivated for activism because they see it as part of their spiritual and moral responsibility (29). In the context of nursing practice, religiosity may influence ethical decision-making and approaches to working with patients. Nurses with strong religious beliefs may be more likely to make decisions based on moral and ethical principles derived from their religion (30).

Our study revealed a positive relationship between the overall NPVS-3 scale score and nurses’ commuting time (*r* = 0.101; *p* = 0.047). Longer commuting time may promote a higher sense of belonging to the profession and a greater value of nursing work. Nurses who spend more time commuting may experience stronger motivation and dedication in their work, seeing it as important and necessary to overcome commuting difficulties (31).

The mean value of extrinsic satisfaction of work (*M* = 35.95, SD = 5.05) is higher than that of intrinsic satisfaction (*M* = 24.42, SD = 3.78), suggesting that nurses may value extrinsic motivators, such as working conditions and salary, more than aspects related to intrinsic motivators. This may be due to the fact that working conditions and salary have a direct impact on their daily lives and well-being, providing them with a sense of financial security and comfort (32, 33). In addition, in the face of heavy workloads and demanding conditions, external factors that improve the work situation are particularly appreciated. Job stability and job security in an occupation subject to high turnover can also play a key role (34). In some health systems and cultures, the emphasis on extrinsic motivators may be stronger and expectations for working conditions and pay higher due to high levels of stress and responsibility. All of the aforementioned factors lead nurses to value extrinsic motivators more, which is reflected in higher average values of extrinsic satisfaction (35).

Analyzing the results of the study, a positive relationship was found between extrinsic satisfaction of work and various factors of nurses such as age, work experience, education and work hours. Similar results regarding the positive relationship between satisfaction and age (as age increases, job satisfaction increases) have been obtained by researchers from India, Saudi Arabia (36, 37). As time passes and work experience increases, nurses may come to appreciate more the external aspects of their jobs, such as employment conditions, pay or career development prospects (38). Higher education also may lead to greater satisfaction with working conditions, perhaps through greater opportunities for career advancement or more favorable contracts with employers (39). Our study found a positive association between on-call length and job satisfaction among nurses, contradicting many previous studies that show a negative association between these variables. Researchers agree that 12 h shift nurses are often associated with negative consequences, such as lower quality of care, job dissatisfaction and higher levels of burnout (26, 40–42).

However, the negative relationship between external satisfaction and marital status indicates that married nurses have lower levels of satisfaction with their working conditions, which may be due to greater family responsibilities or personal life pressures (43). On the other hand, a study by Rashid et al. (44) found a significant relationship between social support from family and job satisfaction. These findings suggest that a supportive environment in family life can significantly affect nurses’ overall life satisfaction and emotional well-being. Family support acts as an important factor in increasing their job satisfaction, confirming that family relationships are crucial to improving the quality of work life in this professional group (44). A positive correlation was found between the HECS subscale on peer relationships and nurses’ education (*H* = 5.638; *p* = 0.048), it is worth noting that this result may suggest that nurses with higher levels of education value interactions and cooperation with other members of the nursing staff more. This may be linked to greater ethical awareness and communication skills, which is conducive to building healthy relationships in the workplace (45, 46).

In our study, a negative correlation was observed between the HECS subscale of relations with physicians and nurses’ participation in the qualification course. Nurses attending courses may be more assertive in expressing their opinions and making decisions, which can create conflict or tension in relationships with physicians (47). In addition, it may also be due to hierarchical changes within the healthcare team, where nurses increase their knowledge and competence, which may be perceived by some doctors as a threat to their authority (48).

Of the 586 people who began filling out the questionnaire, 388 completed the survey, corresponding to a response rate of 66%. This means that 198 participants dropped out before completing the survey. It is worth noting that the reasons for quitting may have been varied, including both factors related to the questionnaire design and the individual characteristics of the participants. For example, those with lower job satisfaction may have been less motivated to complete the survey. This shows research by Blaauw et al. (49) that nurses with low job satisfaction often struggle with job burnout, frustration and lack of commitment. These emotional burdens can result in reduced motivation to engage in additional activities, such as survey participation (49).

### 5.1 Limitations

This study has some limitations. Because the survey began during the SARS-CoV-2 pandemic, it had to be conducted remotely using an online platform. Such remote surveys limit the ability to directly observe behaviors or situations, making it difficult to fully assess nurses’ working conditions. The use of additional qualitative methods, such as interviews or observations, could help to better understand the context of their work and identify subtle factors affecting professional values, the ethical climate in the hospital and job satisfaction.

The study was cross-sectional in nature. It was conducted at a time point, and this makes it impossible to draw causal conclusions—this is also worth emphasizing. The survey sample was conveniently selected, which is a limitation and requires caution when interpreting the results in the context of the entire population of nurses. The study covered only one region of Poland, specifically the Eastern part of the country, which should also be noted. This geographic limitation may lead to a lack of representativeness for the country as a whole, as working conditions and cultural factors may differ in different regions.

The study did not analyze the relationship between gender and professional values, ethical climate of the hospital and job satisfaction, as the male group represented only 4.6% of the study population. Such a small representation of men prevented a reliable and statistically significant comparative analysis between the genders. To study this relationship in the future, research samples can be increased by recruiting more men, using purposive sampling, conducting multi-center studies, and developing analytical methods that take small groups into account. These measures will enable future studies to more accurately analyze differences and similarities in the variables studied between the two genders.

## 6 Conclusion

In a study on the relationship between socio-demographic and professional factors on professional values, job satisfaction and ethical climate among nurses, several significant correlations were observed. Older nurses and those who participated in retraining courses showed lower values related to caring for patients, while higher professional commitment was found in nurses who spent more time commuting to the hospital. In addition, the older nurses with more experience, education and longer work experience showed greater satisfaction with such external aspects of their jobs as pay, working conditions and professional development opportunities. In terms of the ethical climate of the hospital, nurses with more education had better relationships with their colleagues, but may have had difficulties in their relationships with physicians. These findings underscore the need to consider a variety of factors when developing strategies for managing nursing staff and improving the quality of health care. Additional research in this area may contribute to a better understanding of nurses’ professional experiences and identify areas for intervention.

## 7 Implications

Developing effective intervention strategies in the area of the relationship between nurses’ professional values, ethical hospital climate, job satisfaction, and sociodemographic and work environment variables can be a key challenge for managing medical staff and improving the quality of health care. In this context, the development of extensive training and education programs can be an effective intervention strategy. Such programs can include nursing staff and nursing management. Training courses and workshops can be conducted in the form of interactive sessions where participants have the opportunity to discuss, share experiences and practically apply the skills they have learned in the context of nursing work. These programs can focus on developing skills related to ethical decision-making, coping with professional stress and building interpersonal relationships in the workplace.

Supporting leaders and managers in their role of promoting an ethical climate and building a positive work environment can also be an important part of intervention strategies. Managers can be trained to identify and address ethical climate issues, communicate with staff, and effectively manage human resources. By strengthening managerial competencies in the areas of building positive interpersonal relationships and fostering professional values, the management of a medical facility can contribute to creating an open, trusted and productive work environment.

An important aspect of the intervention strategy is also to create open channels of communication that will enable medical employees to express their concerns, suggestions and participate in decision-making regarding the ethical climate and professional values. Open and direct communication can help build trust, increase professional engagement and improve staff-management relations. In practice, this means creating mechanisms that allow employees to participate in decisions about work organization, assess changes in the work environment, and adjust intervention strategies to meet the current needs of medical staff.

However, the implementation of these strategies requires a holistic approach and continuous monitoring of the effects to adapt the activities to changing needs and working conditions.

## Data Availability

The raw data supporting the conclusions of this article will be made available by the authors without undue reservation.
